# Application of Point-of-Care Ultrasound for Family Medicine Physicians for Abdominopelvic and Soft Tissue Assessment

**DOI:** 10.7759/cureus.9723

**Published:** 2020-08-13

**Authors:** Sarah E Frasure, Elizabeth Dearing, Morgan Burke, Maria Portela, Ali Pourmand

**Affiliations:** 1 Department of Emergency Medicine, George Washington University School of Medicine and Health Sciences, Washington DC, USA; 2 Medicine, George Washington University School of Medicine and Health Sciences, Washington DC, USA; 3 Primary Care, George Washington University School of Medicine and Health Sciences, Washington DC, USA

**Keywords:** point-of-care ultrasound, primary care, internal medicine, bedside ultrasound

## Abstract

Point-of-care ultrasound (POCUS) improves both the sensitivity and specificity with which clinicians can make a variety of diagnoses at the bedside from abdominal aortic aneurysm to kidney stones. In outpatient clinics, urgent care centers, and emergency departments, where ultrasound imaging may be delayed by hours or even days, the use of POCUS can be very helpful. We believe that POCUS facilitates both the triage of patients and provides diagnostic information quickly. We hope to advance the use of POCUS in the primary care setting and have reviewed six sonographic topics where we believe ultrasound can be of immense assistance to the physician in the outpatient setting.

## Introduction and background

Clinical ultrasonography, or point-of-care ultrasound (POCUS), has changed greatly over the past two decades - both in the size and portability of the machine and in the range of clinical applications. Emergency physicians (EP) and critical care physicians have increasingly integrated POCUS into the clinical care of patients, using ultrasound machines at the bedside. Family Medicine Physicians have traditionally used ultrasound most often during obstetric related care and when available for musculoskeletal injuries. POCUS improves both the sensitivity and specificity with which clinicians can make a variety of diagnoses from abdominal aortic aneurysm to testicular torsion [1-3]. Using a compact ultrasound machine at the patient's bedside also improves the speed at which physicians are able to make a clinical diagnosis, which in turn hastens both initial treatment and disposition decisions [4]. In rural clinics and busy emergency departments, where comprehensive ultrasound imaging may be delayed by several hours (or days in the outpatient setting), the use of POCUS is particularly salient [4]. Certainly, POCUS facilitates both the triage of patients and provides diagnostic information quickly [4,5]. Furthermore, POCUS is more cost-effective than other imaging modalities, such as computerized tomography (CT) or magnetic resonance imaging (MRI). In today’s world of rising healthcare costs, cost-effective care is increasingly relevant. Ultrasound is also a safe imaging option compared to CT or X-ray, as there is no ionizing radiation exposure.

POCUS was first adapted by EPs for the rapid identification of life-threatening cases of cardiac or pulmonary pathologies [6,7]. The quick identification of the diagnosis then allows EPs to administer lifesaving treatment for conditions such as traumatic intra-abdominal bleeding, spontaneous pneumothorax, ruptured ectopic pregnancy or massive pulmonary embolism. Guidelines for core ultrasound competencies that have been established by the American College of Emergency Physicians include trauma, pregnancy, abdominal aortic aneurysm, cardiac, biliary, urinary tract, thoracic, deep vein thrombosis, ocular, and soft tissue [8,9]. In the last few years, the American Academy of Family Physicians (AAFP) published recommended guidelines for family medicine residency programs to implement a curriculum in POCUS. Since then, recent research has advocated for the case of expanding the POCUS use and adaption in Family Medicine. In addition, we believe a next step in the adoption of this helpful clinical diagnostic tool would be for it to be considered as a competency in the primary care residencies curriculum as well. We standby the opinion of others that there is great potential for the applicability of POCUS in less acute care settings, such as in family medicine and primary care clinics [4,8-11]. Specifically, POCUS can not only improve the sensitivity of primary care physicians in detecting illnesses, but also improves the specificity at which they do so [8,10]. For instance, thoracic POCUS has a higher degree of sensitivity and specificity for pneumonia compared to classic auscultation, and does so with the same level of precision as a chest X-ray or CT scan [10,12]. POCUS can also be used by primary care physicians to evaluate for the presence of an abdominal aortic aneurysm, a pleural effusion, pulmonary edema, a proximal leg deep vein thrombosis, and differentiation of skin and soft tissue infections [10,13,14]. Certainly, POCUS can also assist primary care physicians when making decisions about referrals, screening procedures, and general health checkups [15]. Our aim is to advance the use of POCUS imaging in the primary care setting. Thus, we will review six sonographic topics where we believe ultrasound can be of immense assistance to the physician in the outpatient setting. 

## Review

Ultrasound probe selection

Standard imaging orientation principles must be understood to obtain quality images for clinical use. All ultrasound transducers contain a marker located on one side of the probe, although the actual appearance of this marker changes depending on the manufacturer of the machine/probe. This marker on the transducer corresponds to a marker on the ultrasound screen. In general, the marker on the screen will be located in the upper left corner. The marker on the probe should be aimed at the patient’s head or to the right side of the patient’s body. This allows for appropriate orientation and can also guide the physician to adjust probe positioning on the patient in order to obtain an ideal image [16].

There are high and low frequency transducer for ultrasound machines. The frequency of the ultrasound transducer determines both resolution and depth of images. The high frequency transducers (such as a linear probe) produce the highest resolution images. However, the depth of penetration is limited with high frequency probes. For example, the linear probe provides the excellent detail of superficial structures like skin, muscles, and bones - yet it can only examine up to a depth of 6 cm. The lower frequency probes (examples include the phased array and curvilinear transducers) penetrate deeper into the body (20-30 cm) but the images are less detailed, and, thus, have a lower resolution. As a result, choice of transducer is determined by the depth of the object of interest [16]. For example, a high frequency transducer should be utilized when assessing soft tissue for an abscess, while a low frequency probe can help the physician look for an abdominal aortic aneurysm (AAA).

Soft tissue infections

POCUS can differentiate between an abscess and cellulitis. Abscesses are pyogenic infections, which generally begin as a localized cellulitis but develop into a pocket of purulent material, which requires incision and drainage for definitive treatment. When attempting to decide whether to perform an incision and drainage of a suspected abscess within a larger area of cellulitic-appearing tissue, the physician may not always be able to palpate a distinct area of fluctuance. In such cases, POCUS can assist the physician by identifying any fluid collections underlying areas of cellulitis.

**Figure 1:**
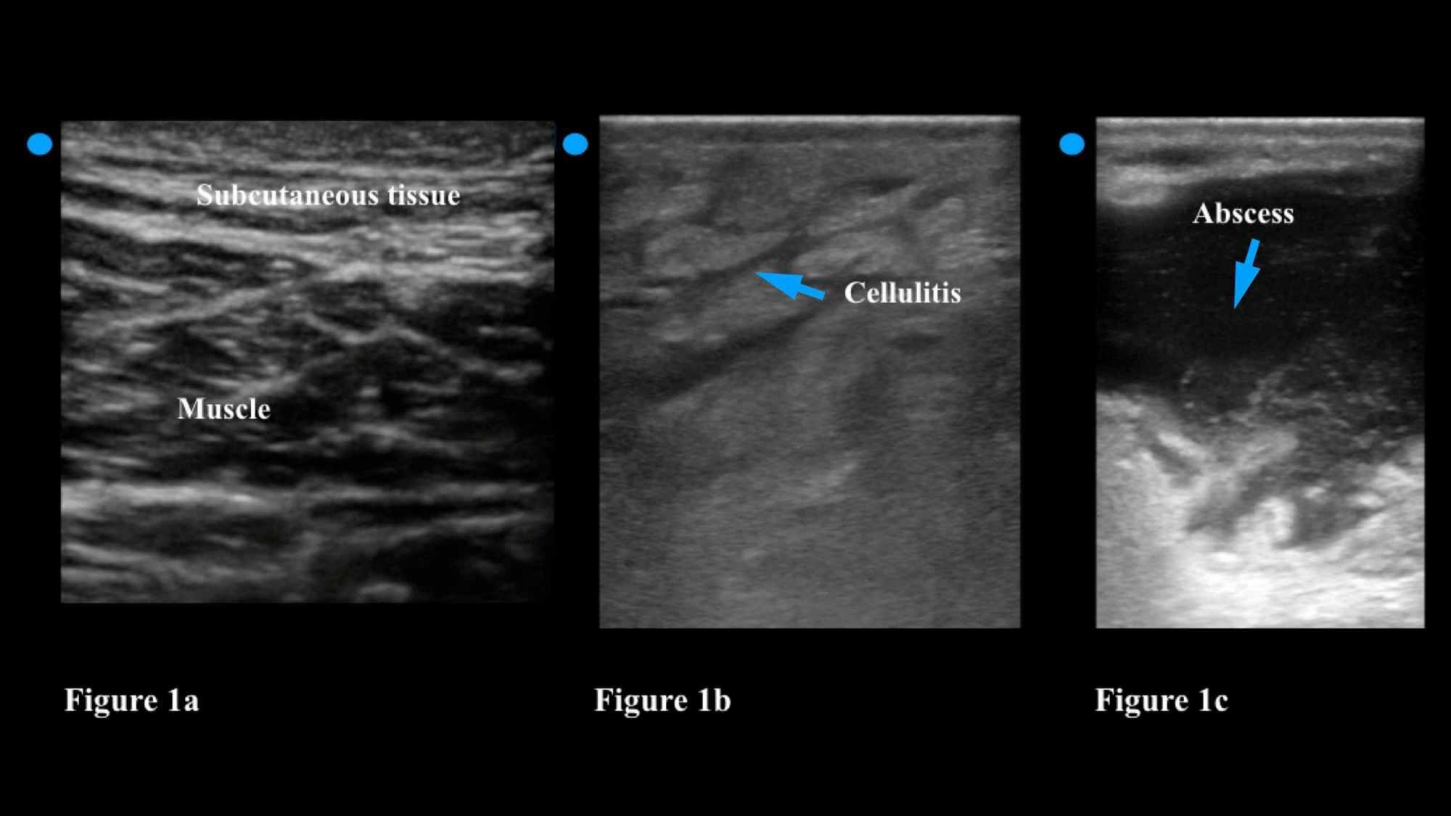
Figure 1a: Normal soft tissue ultrasound demonstrates thin layers of subcutaneous tissue overlying the muscle tissue. Figure 1b: Cellulitis is noted as thickening of the epidermis and subcutaneous layers; it is often described as ‘cobblestoning.’ Figure 1c: An abscess has a circumferential and usually irregularly shaped wall; it is filled with hypoechoic fluid and will often have echogenic particles floating within it.

The physician should choose a linear transducer when looking for the presence of an abscess. The linear transducer has a high frequency (7.5-10 MHz), which provides excellent resolution of soft tissue. The physician should move the transducer slowly over healthy skin and then move across the affected soft tissue in two planes [17]. Compared to healthy tissue cellulitis can be recognized on ultrasound by a distinct thickening of the epidermis and subcutaneous layers (Figure 1a). The appearance of ‘cobblestoning’ is frequently described; the physician will note thin bands of hypoechoic or anechoic (dark) fluid among layers of hyperemic (white), inflamed subcutaneous tissue (Figure 1b). An abscess, on the other hand, has a distinct circumferential, and usually irregularly shaped wall, that is filled with hypoechoic (dark) fluid but will usually have echogenic particles floating within it (Figure 1c). By gently pushing the transducer down the physician can compress the abscess. The maneuver will lead to the movement of these particles with the fluid collection - this is called the ‘squish sign’ and distinguishes an abscess from a lymph node, a vessel, or a soft tissue mass [18]. The ultrasound can also be used in real-time to guide the incision if the abscess is deep or near a vascular structure.

Foreign bodies

Injuries that lead to the retention of subcutaneous foreign bodies are common in adults and children. Unfortunately, however, foreign bodies are often difficult to detect by physical examination. Although radiographs can help the physician locate opaque foreign bodies (metallic pins, staples, glass), plain film imaging cannot visualize plastic or wooden objects (splinters, thorns, etc). POCUS, however, helps the physician to localize both radiopaque and radiolucent foreign bodies, and also aid in removal by showing the physician where to make the incision and how deep the foreign body is. Additionally, ultrasound does not have the radiation of the CT or the large cost of MRI. For these reasons, ultrasound has been recommended as the initial imaging modality for primary detection or as adjunct in cases where plain radiographs are negative and concern for radiolucent objects remains [19,20].

**Figure 2:**
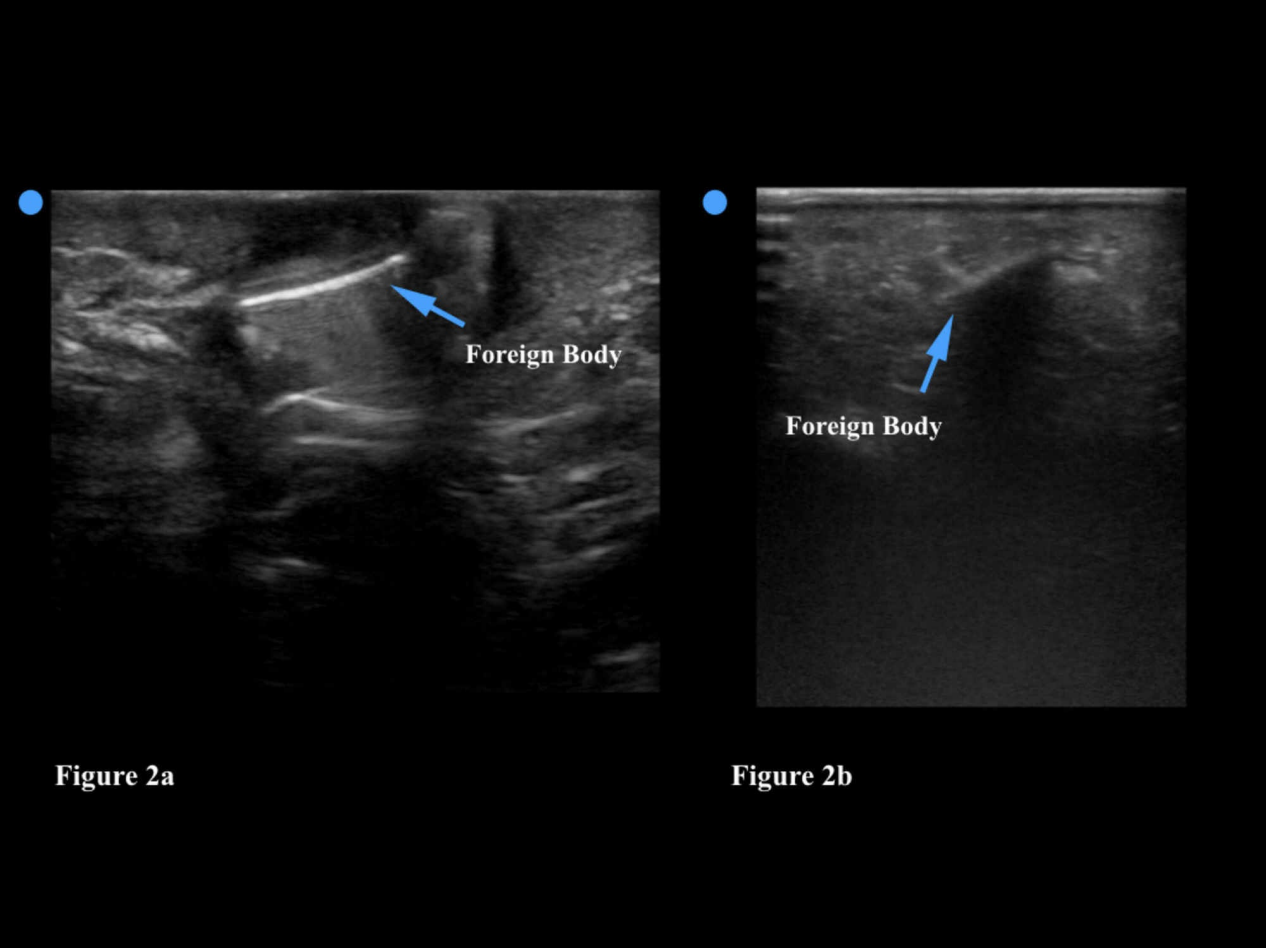
Figure 2a: This foreign body is hyperechoic (bright) and has a distinct reverberation artifact. Figure 2b: This foreign body demonstrates a shadowing artifact.

Evaluation for a foreign body should be performed using a linear transducer. The linear transducer transmits high frequency sound waves and produces the highest resolution images. High resolution images allow the physician to visualize more detail; since the vast majority of foreign bodies are located in the subcutaneous tissue, the greater depth afforded by the curvilinear or phased array transducers is not essential. As with any POCUS, the area of interest (in this case where the patient feels pain, possibly attributed to an embedded foreign body) should be thoroughly scanned in two orthogonal planes (e.g. longitudinal and transverse). If a foreign body is identified, the physician can subsequently use the ultrasound to determine the size, depth, and overall appearance of the object. Foreign bodies that are embedded in soft tissue generally appear hyperechoic (bright) on ultrasound. Foreign bodies can also often be detected by the acoustic artifact that they create - which include a reverberation artifact as seen in Figure 2a and a shadowing artifact (Figure 2b).

POCUS can also help guide the removal of a foreign body in the subcutaneous tissue. The provider can inject a small amount of lidocaine around the foreign body to loosen it from the tissue and facilitate its removal - both of these procedures can be performed under ultrasound guidance. Finally, the physician can use ultrasound to ensure that no small piece of the foreign body has remained in the soft tissue once the procedure is complete. The physician should be aware that the introduction of air into the site (whether secondary to the injury itself or due to wound exploration) could cause artifacts that may obscure the foreign body on ultrasound. Furthermore, the physician may fail to identify a foreign body if he/she does not recognize the artifact that some foreign bodies create for the sound waves or if the foreign body is too close to another structure, such as bone, that may obscure the artifact [21]. Deep or very small foreign bodies may also be harder to visualize and can occasionally be missed on ultrasound [22]. As with all sonographic techniques, the more soft tissue ultrasound scans the physician does, the more comfortable he/she will be with this process, and the less likely he/she will miss a foreign body. 

Gallbladder

**Figure 3:**
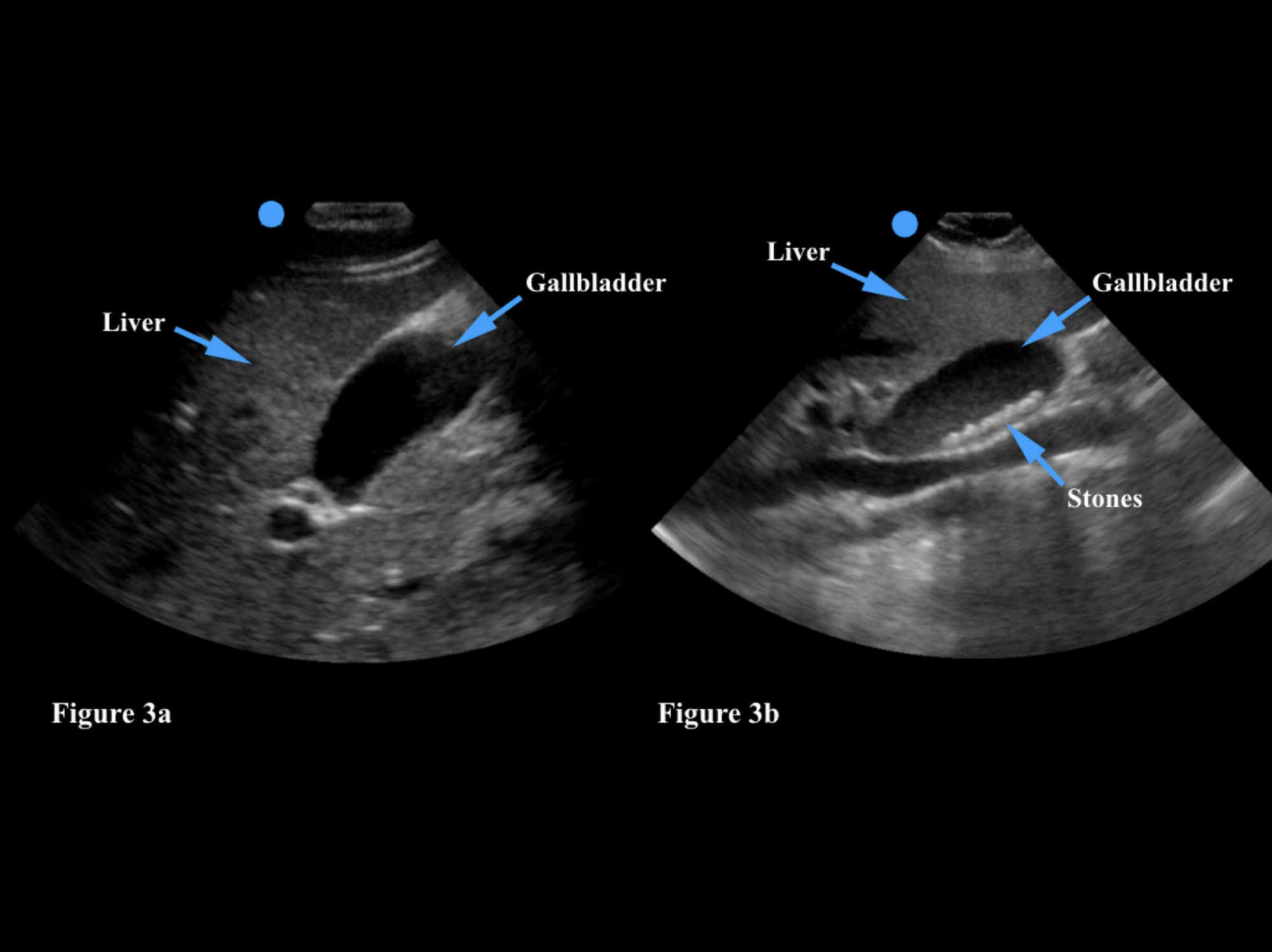
Figure 3a: The gallbladder is a hypoechoic (dark) structure which generally overlies the liver. Figure 3b: Gallstones are generally hyperechoic (white) and will create a shadow artifact.

Gallstones affect approximately 10% of the population and are more likely to occur in middle-aged women, though the prevalence increases with age irrespective of gender. A family history of gallstones and obesity are also well-described risk factors [23]. POCUS is routinely used to evaluate for the presence of gallstones in patients with post-prandial right upper quadrant pain, nausea, and/or vomiting in the emergency department. The sensitivity and specificity of abdominal ultrasound for the assessment of cholelithiasis exceeds 95% with gallstones greater than 1.5 mm in size [24]. The physician should choose either a phased array or curvilinear transducer in order to ensure adequate depth of imaging. The gallbladder should be viewed in two planes; it is a hypoechoic structure that is usually found either overlying or medial to the liver (Figure 3a). The physician should put color flow or doppler imaging on the gallbladder to ensure that he/she has not mistakenly identified a vascular structure (such as the aorta or inferior vena cava) as the gallbladder. Gallstones are generally hyperechoic (white) on ultrasound and should move around within the gallbladder if the physician has the patient rotate from the supine position to the left lateral decubitus position (Figure 3b). In addition, a gallstone should shadow posteriorly on ultrasound imaging as it prevents the ultrasound beam from transmitting sound. In contrast, gallbladder polyps do not shadow and should not move with a change in patient position as they are fixed to the gallbladder wall. Sonographic findings of acute cholecystitis include the presence of a gallstone and abdominal pain when the transducer is placed over the gallbladder (also known as a positive Murphy sign), gallbladder wall thickening (>3mm), and fluid around the gallbladder (pericholecysitic fluid) [24]. Of note, the physician may also occasionally find gallstones or polyps incidentally while performing an unrelated study, such as a renal or aorta ultrasound. In this case, the patient should be made aware of the sonographic findings and educated regarding symptoms of cholelithiasis/cholecystitis.

Aorta

Patients with an AAA may present to their primary care physician with complaints of abdominal, groin, or back pain - symptoms that are often attributed to renal colic or muscle spasm. POCUS of the abdominal aorta can be quickly performed in order to rule out AAA before making an alternate diagnosis. Ultrasound has both a high sensitivity (97.5%-100%) and specificity (94.1%-100%) for detecting AAA [25]. Furthermore, even a one-time screening of men over the age of 65 can reduce AAA-related mortality, and the need for immediate repair. Risk factors include male gender, a history of smoking, and a family history of AAA [26]. POCUS is commonly performed in older patients who present to the emergency department with hypotension, abdominal/back pain, or syncope, to assess for the presence of a AAA. We believe this screening tool could be incredibly helpful for primary care providers as well.

**Figure 4:**
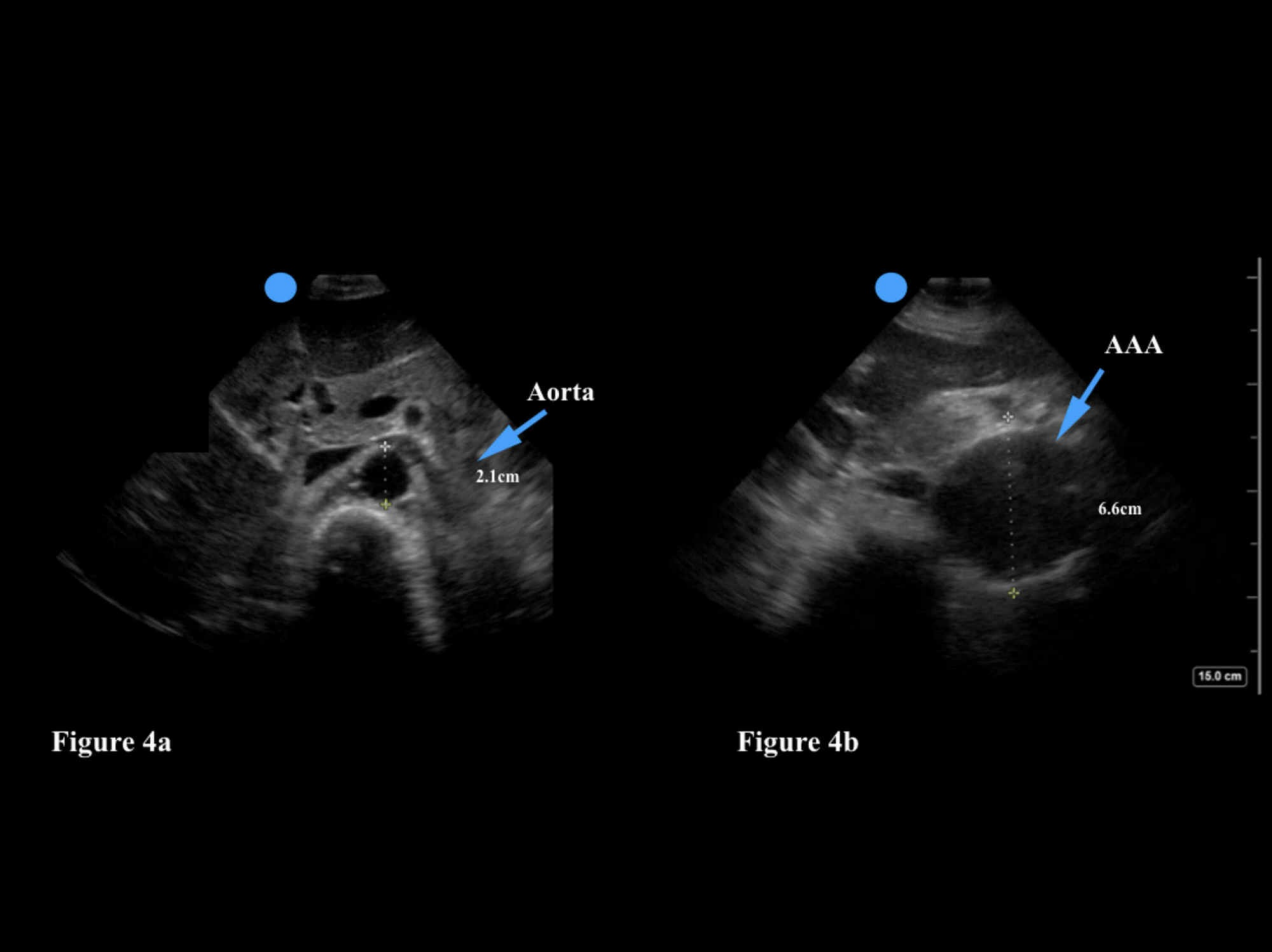
Figure 4a: A normal aorta should have a diameter <3 cm. Figure 4b: This abdominal aortic aneurysm is 6.6 cm in diameter.

Ultrasound examination of the abdominal aorta should be performed with a low frequency transducer; the curvilinear and phased array transducers are both appropriate choices. The physician should scan the aorta in two planes - examining the abdominal aorta from its proximal section to the bifurcation of the aorta into the iliac arteries (Figure 4a). The abdominal aorta can be distinguished from the inferior vena cava (IVC) by three characteristics: the aorta has a thicker wall, is a pulsatile vessel, and will not compress with firm pressure of the transducer [27]. The physician can also use color doppler to identify arterial flow in the aorta. 

Emergency physicians will rule in an AAA in patients with abdominal/back pain and/or syncope/hypotension if the diameter is >3 cm or if either of the iliac arteries are greater than 1.5 cm in diameter. If the patient is hemodynamically stable, he/she will generally undergo CT imaging to assess for an aortic leak (Figure 4b). If the patient is hemodynamically unstable, however, an emergency vascular surgery consultation is obtained to consider immediate operative treatment. Primary care physicians can use POCUS to assess their older patients for the presence of a AAA and even assess the size of a AAA during yearly follow-up visits. Of note, if the physician is unable to view the abdominal aorta in its entirety a AAA may be missed. Poor visualization of the aorta may occur as a result of body habitus (obesity can make sonography more difficult given the greater depth needed for the ultrasound waves to travel), or overlying bowel gas [27]. Constant pressure to the abdomen with the transducer can help to move bowel gas out of the way and bring the aorta into view. This technique may be of limited use, however, if the patient has abdominal pain and does not tolerate the extra pressure.

Kidneys and bladder

Kidney stones affect approximately 5% of the general population [28]. Patients may complain of acute back or groin pain, hematuria, and nausea or vomiting. Risk factors include male gender, a family history of kidney stones, dehydration, and a high protein diet. Point-of-care renal ultrasound is routinely used in the emergency department to assess for hydronephrosis which serves as a marker of a kidney stone in the renal collecting system. Stones are usually stuck at the uteropelvic junction (UPJ) and the ureterovesicular junction (UVJ); these are the narrowest areas within the ureter. Although the physician may not always identify the culprit kidney stone on ultrasound, the presence of hydronephrosis indicates a downstream obstruction in the ureter. Compared to CT imaging, ultrasound reduces time to diagnosis and subsequently hastens appropriate treatment.

**Figure 5:**
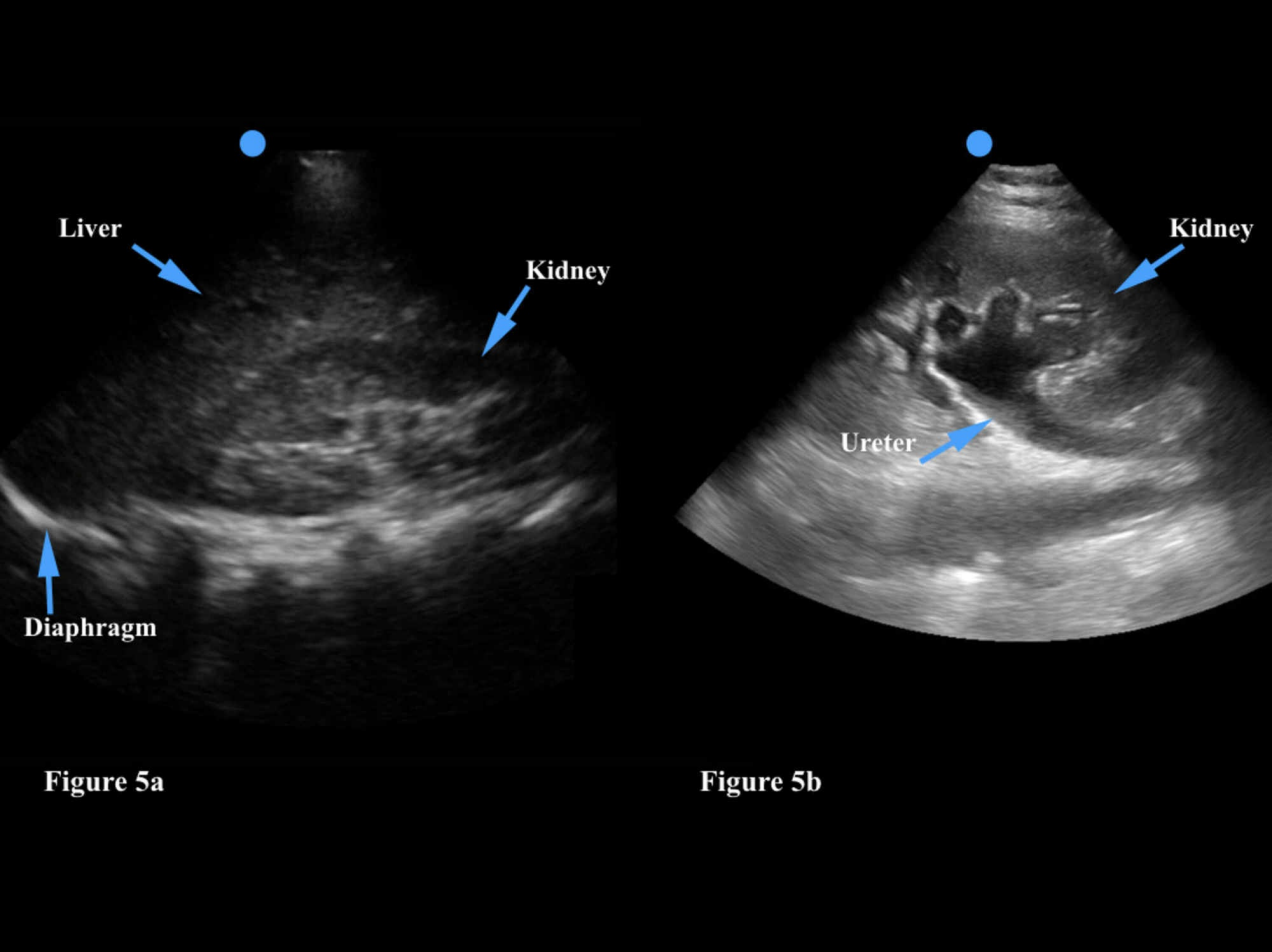
Figure 5a: The kidney is generally found inferior to the liver. The renal pelvis is hyperechoic. Figure 5b: Hydronephrosis is identified as a large hypoechoic (dark) area in the renal pelvis, which is normally hyperechoic.

The physician may choose either the curvilinear or phased array transducer - both provide adequate depth to examine the kidneys. As with any organ of interest, the kidneys should ideally be visualized in two planes (transverse and longitudinal) in order to perform a complete examination [29]. The right kidney is generally much easier to visualize than the left kidney as the liver provides an excellent acoustic window in contrast to the much smaller spleen (Figure 5a). Hydronephrosis is identified as a large hypoechoic (dark) area in the renal pelvis, which is normally hyperechoic (Figure 5b). The physician should visualize this abnormal finding in two planes. Unilateral hydronephrosis is highly suggestive of a kidney stone. Of note, patients with renal colic who are severely dehydrated and unable to keep fluids down due to persistent emesis may not have hydronephrosis on the initial ultrasound - in this case the kidneys can be reimaged after an intravenous fluid bolus is provided.

The physician can also utilize POCUS to assess bladder volume in patients who complain of difficulty urinating, urinary hesitancy, urinary frequency, or overflow incontinence. More than one-half of noninstitutionalized women and more than one-quarter of noninstitutionalized men > 65 years of age report urinary leakage [30]. Furthermore, urinary retention is associated with urinary tract infections, bladder overdistention, and higher mortality rates. The most common cause of acute urinary retention is bladder over-distention after undergoing a surgical procedure that necessitated general anesthesia [31].

**Figure 6:**
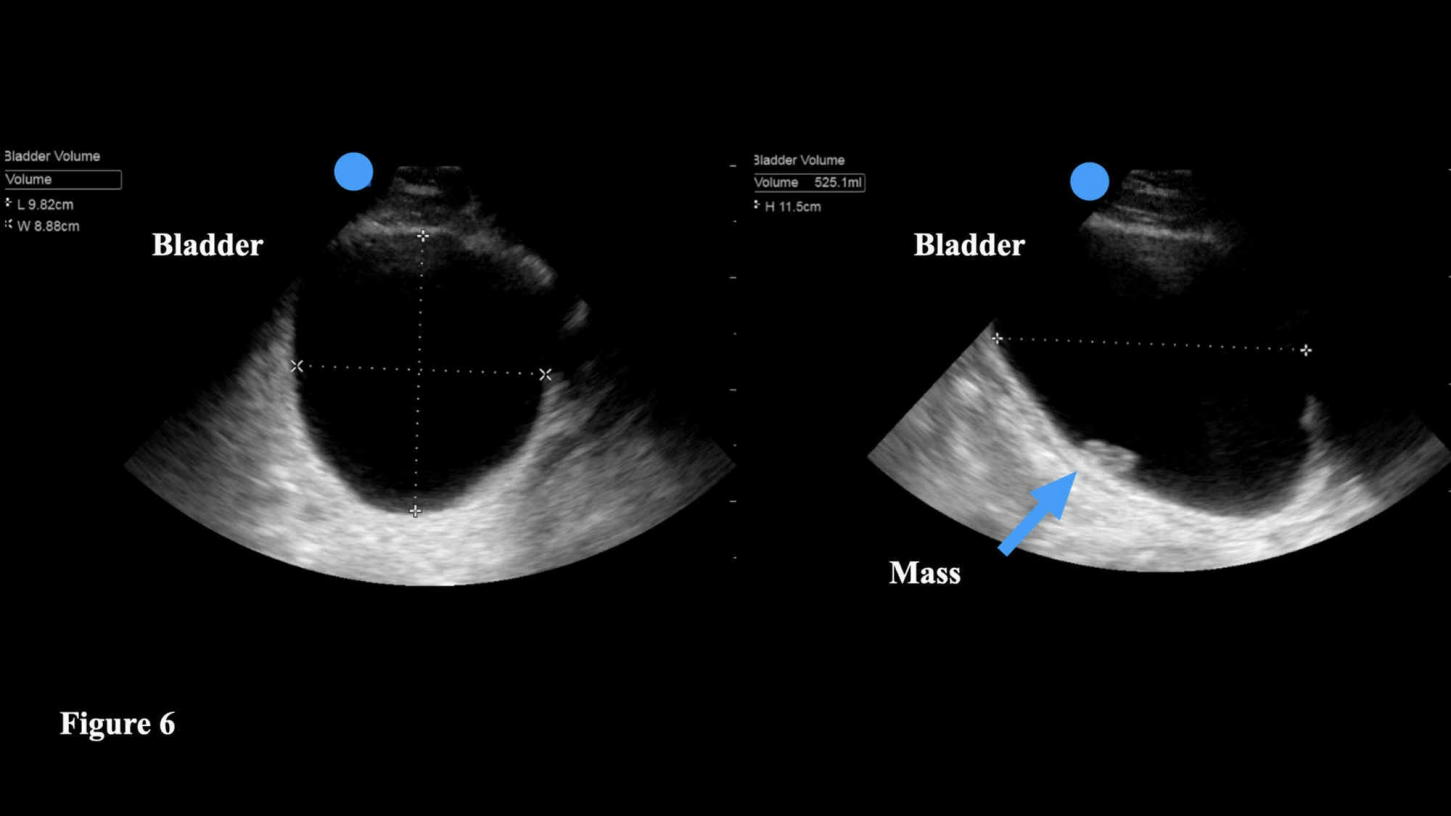
The height of the bladder (h) can be found by measuring the longest sagittal (longitudinal) plane of the bladder. By turning the transducer into a transverse view, the physician can measure the maximal length (l). In the same plane, measure the maximum width (w). The majority of ultrasound machines will automatically calculate the bladder volume when these three measurements are saved. This image also shows an incidentally found bladder mass (arrow).

POCUS can help the physician to quantify the amount of urine in the bladder if the patient is unable to void, as well as a post-void residual urine volume. The physician should employ either the curvilinear or phased array transducer to ensure adequate depth. The bladder should be imaged in two planes - longitudinal and transverse [32]. The majority of ultrasound machines have a bladder volume software package that automatically provides a bladder volume after the physician takes three distinct measurements. Specifically, the height of the bladder (h) can be found by measuring the longest sagittal (longitudinal) plane of the bladder. Then, by turning the transducer into a transverse view, the physician can measure the maximal length (l). In the same plane, measure the maximum width (w) (Figure 6). Should the ultrasound machine not have a means to calculate the bladder volume, the physician can use the following formula - volume = 0.7 x h x l x w.

Obstetrics-gynecology

Point-of-care transabdominal ultrasound is already widely used in both the emergency department and some urgent care settings to assess for the presence of an intrauterine pregnancy (IUP) in patients with abdominal pain and/or vaginal bleeding with a positive pregnancy test. When an IUP is not visualized expedited transvaginal sonography can be ordered to look for an early IUP versus an ectopic pregnancy [33]. In addition, physicians can use transabdominal imaging to assess the fetal heartbeat and obtain an estimated gestational age by measuring the length of the fetus. The provider should be cognizant of the theoretical risk of tissue heating with prolonged use of ultrasound, which is most salient when assessing for early pregnancy. Thus, when performing transabdominal obstetric ultrasound, the physician should abide by the ALARA (as-low-as reasonably-achievable) principle and minimize the duration of imaging [34]. 

**Figure 7:**
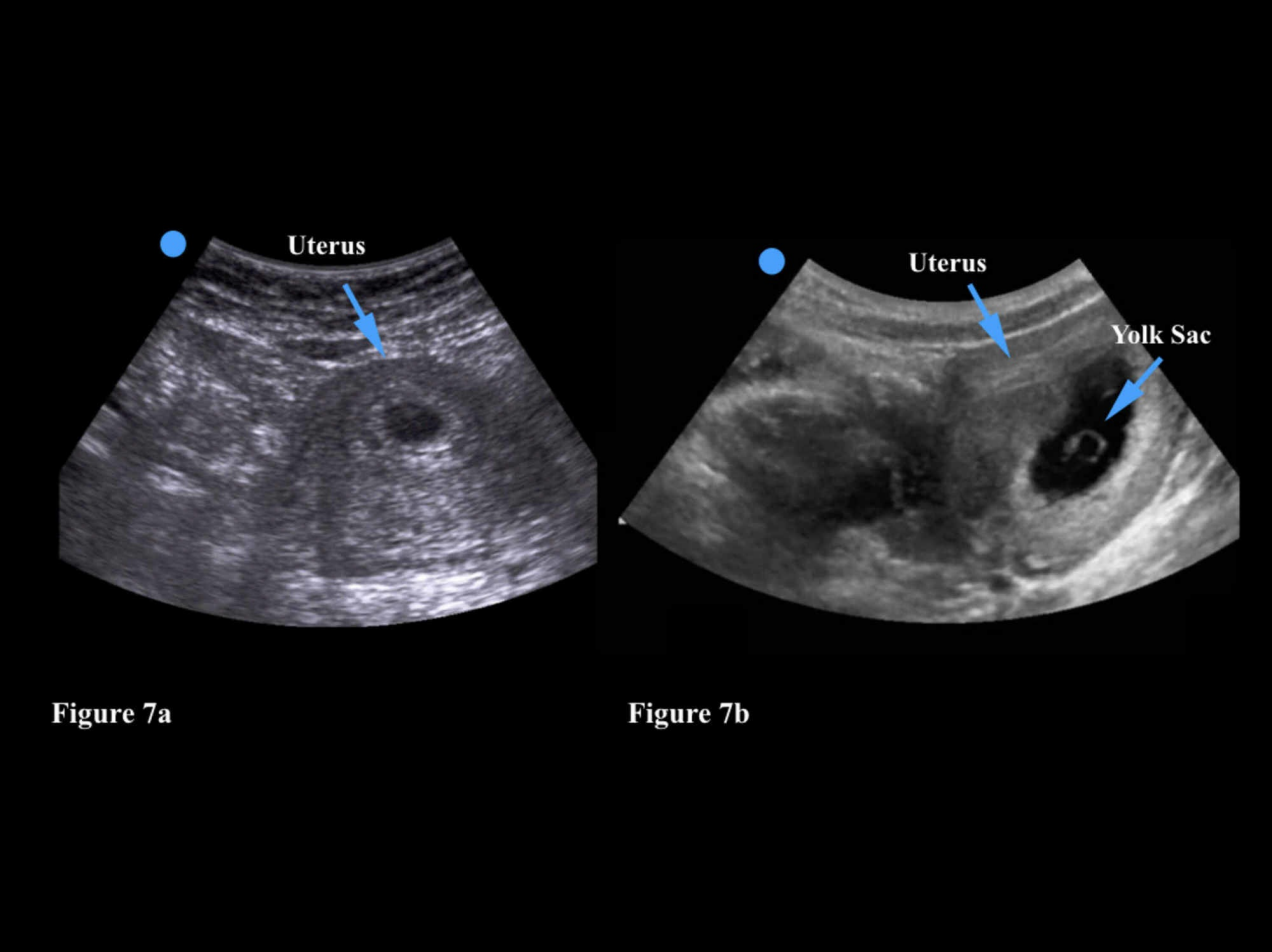
Figure 7a: An empty gestational sac within a uterus may signify a very early pregnancy but can also indicate an ectopic pregnancy. Figure 7b: This gestational sac has a circular yolk sac (often called the diamond ring sign) which is sonographic evidence of an intrauterine pregnancy.

In the primary care setting, point-of-care transabdominal ultrasound could be very helpful in the identification of an IUP in pregnant patients. The physician can use either the curvilinear or phased array transducer as both options provide adequate imaging depth. In very thin patients, however, the linear transducer may also a reasonable choice. The physician should examine the uterus in both the longitudinal and transverse planes in order to ensure that the pregnancy is contained within the uterus. An empty gestational sac may signify a very early pregnancy (<4 weeks) but can also indicate an ectopic pregnancy (Figure 7a). An IUP is generally visualized at 5-6 weeks on transabdominal ultrasound with the identification of a gestational sac and a yolk sac or fetal pole (Figure 7b). Patients with a positive pregnancy test and a transabdominal ultrasound that demonstrates an empty gestational sac should be referred for prompt transvaginal imaging. These patients will also need serial human chorionic gonadotropin (hCG) measurements to ensure that the pregnancy is progressing normally.

At six weeks the physician should also be able to measure a fetal heart rate using transabdominal ultrasound imaging. By applying M-mode, which calculates motion over time, the physician can determine an accurate heartbeat. A fetal heart rate that is <100 at six weeks of gestation is highly concerning for a failed pregnancy and should prompt an urgent obstetrics consult [35]. In addition to a FHR measurement, the physician can calculate an estimated gestational age. In the first trimester the crown-rump length can be easily acquired. In the second and third trimesters the physician should measure the femur length, head circumference, or biparietal diameter, in order to determine the gestational age.

Of note, the physician may occasionally visualize an intrauterine device (IUD) while using transabdominal ultrasound to evaluate a patient. IUDs are hyperechoic, and, thus, highly reflective, and have a shadow artifact. Of note, the exact position of an IUD within the uterus is much more accurately visualized with transvaginal sonography [36]. Thus, patients with abdominal pain and/or vaginal bleeding who have an IUD should ideally undergo transvaginal imaging to assess for IUD malposition. 

Finally, physicians can use POCUS to assess the placement of a subdermally implanted contraceptive rod. This skill is particularly useful when external palpation of the contraceptive rod is not possible [37]. Similarly to the sonographic assessment of cellulitis, an abscess, or a retained foreign body, the physician should choose the linear transducer which provides excellent subcutaneous and musculoskeletal image quality. 

 There are many other advanced imaging options for physicians who wish to learn more about POCUS. In the emergency department, we routinely perform bedside cardiac ultrasounds to assess for a pericardial effusion, reduced ejection function, and evidence of right ventricular strain that may indicate a pulmonary embolus. The Focused Assessment of Sonography in Trauma (FAST) exam allows physicians to quickly evaluate trauma patients for intraabdominal bleeding, pneumothorax, hemothorax, and traumatic pericardial effusions. Thoracic ultrasound can also be a very useful adjunct in dyspneic patients - the physician may find evidence of pneumonia, pleural effusions, B-lines (which are indicative of pulmonary edema), or even a spontaneous pneumothorax. In addition, emergency physicians can perform ocular ultrasounds to look for retinal detachment, lens dislocation, and retinal and/or vitreous hemorrhage in patients who present with blurred vision or ocular trauma.

The future of ultrasound

POCUS reduces patient length of stay in the emergency department [38]. While exact numbers vary across emergency departments, reduction in length of stay ranged from 26 minutes to two hours [39,40]. This reduction in the length of stay is most significant during evening, overnight, and weekend visits [38,41]. Physicians who are interested in learning more about POCUS may be worried about their ability to learn how to use an ultrasound machine and correctly interpret images. Yet, POCUS has been shown to improve physician clinical diagnostic decision making after receiving a two-hour course [42]. Another study which evaluated the use of POCUS among primary care physicians in the United States Army determined that the time to physician education was not an obstacle to the successful implementation of this new technology [43]. While POCUS is a core competency for all emergency medicine residents, other specialties have been slower to adopt it into clinical care. As ultrasound machines become increasingly affordable and smaller in size, however, the use of POCUS is expanding into other areas including primary care, family medicine, intensive care, and internal medicine [44]. In addition a number of medical schools have begun to incorporate ultrasound learning during anatomy sessions. Medical students are also learning how to place ultrasound-guided peripheral intravenous lines, and perform ultrasound-guided procedures such as paracentesis, thoracentesis, and lumbar punctures. 

Note: All images are courtesy of SE Frasure MD and E Dearing MD.

## Conclusions

For physicians who wish to learn more about the use of POCUS, there exists a variety of courses that take place across the United States of America each year. If the physician works at an academic institution, there may also be local courses run by the emergency medicine faculty who are generally responsible for teaching POCUS to their medical students, residents, and ultrasound fellows. A variety of national courses across the United States also exist - they usually run for 2-3 days and include both lectures and hands-on ultrasound teaching to familiarize attendees with image acquisition and interpretation at the bedside. Most of these courses also provide continuing medical education (CME) to the attendees. Should residents or attending physicians wish to dedicate more time to learn how to use ultrasound at the bedside, POCUS fellowships are also available. Currently, there are 138 adult and pediatric emergency medicine ultrasound fellowship programs in the United States of America and per our knowledge less than 15 Primary Care POCUS fellowships. The year-long program provides the fellow with the necessary skills to perform advanced ultrasound imaging, ultrasound teaching, research, and administration.

In conclusion, we believe that the integration of POCUS into the clinic/outpatient setting has the potential to provide physicians with an incredibly useful bedside tool to help with diagnostic decision making and expedition of medical treatment.
